# Life skills as a behaviour change strategy in the prevention of HIV and AIDS: Perceptions of students in an open and distance learning institution

**DOI:** 10.1080/17290376.2017.1374878

**Published:** 2017-09-21

**Authors:** B.J. Mohapi, E.M. Pitsoane

**Affiliations:** ^a^ Senior Lecturer, Department of Social Work, University of South Africa, Pretoria, South Africa; ^b^ Acting Head, Student Counselling, Midlands Region, University of South Africa, Rustenburg, South Africa

**Keywords:** life skills, HIV/AIDS, students, youth, HIV prevention, Aptitudes de vie, VIH/SIDA, Etudiants, Les jeunes, La prévention de l’infection à VIH

## Abstract

The prevention of HIV and AIDS, especially amongst young people, is very important, as they are the future leaders. South Africa carries a high burden of the HIV and AIDS disease, and efforts at the prevention of the disease need to be intensified. University students are also at risk, and prevention efforts need to be intensified to ensure that students graduate and enter the world of work to become productive citizens. Failure to pay attention to preventative behaviour amongst university students may have negative socio-economic consequences for the country. The paper presents a quantitative study undertaken amongst students at the University of South Africa, an Open and Distance Learning Institution in South Africa. The aim of the study was to explore the perceptions of students regarding life skills as a behaviour change strategy at Unisa. The study was conducted in the three regions of the University: Midlands region, Gautengregion and Limpopo region. Data were collected by means of self-administered questionnaires and were analysed by using the Statistical Programme for Social Sciences. The findings revealed that students have a need to attend life skills workshops, which are facilitated by trained student counsellors since they believe that the life skills training will assist them to be assertive and practise behaviours which will not make them vulnerable to the HIV and AIDS infection.

## Résumé

La prévention du VIH et du SIDA, en particulier parmi les jeunes, est très importante car ils sont les meneurs de demain. L’Afrique du Sud porte un lourd fardeau de maladies liées à l’infection à VIH/SIDA, et les efforts destinés à prévenir l’infection doivent être intensifiés. Les étudiants universitaires sont également à risque et il faut intensifier les efforts de prévention pour s’assurer que les étudiants obtiennent leurs diplômes et s’insèrent dans le monde du travail pour devenir des citoyens productifs. Ne pas accorder d’importance aux comportements préventifs parmi les étudiants universitaires peut avoir des conséquences socio-économiques négatives pour le pays. L’article présente une étude quantitative menée auprès d’un groupe d’étudiants de l’Université d’Afrique du Sud, une Institution d’Enseignement Ouvert et à Distance en Afrique du Sud. Le but de l’étude était d’explorer les perceptions des étudiants concernant les aptitudes de vie comme stratégie de changement de comportement à UNISA. L’étude était menée dans trois des campus régionaux de l’Université : le campus de Midlands, le campus de Gauteng et le campus de Limpopo. Les données ont été recueillies au moyen de questionnaires autogérées et ont été analysées en utilisant la Programme Statistique pour les Sciences Sociales (PSSS). Les résultats ont révélés que les étudiants ont besoin de participer aux ateliers des aptitudes de vie qui sont facilités par des conseillers formés car ils croient que la formation aux compétences de vie les aidera à s’affirmer et pratiquer des comportements qui ne les rendront pas vulnérables à l’infection à VIH/SIDA.

## Introduction

The prevention of HIV and AIDS in South Africa is of paramount importance if the battle against the spread of HIV and AIDS is to be won. The National Planning Commission (2011) asserts that South Africa in 2007 represented 0.07% of the world’s population, but had 17% of the global number of HIV infections. The Statistics South Africa Report (2013) stated that the total number of persons living with HIV in South Africa increased from an estimated 4 million in 2002 to 5.26 million by 2013. This means that in 2013 an estimated 10% of the total population of South Africa was HIV positive. The report further asserts that for adults aged 15–49 years, an estimated 15.9% of the population is HIV positive. This paints a very grim picture regarding HIV and AIDS infections in South Africa, and there is a need for a concerted effort if the spread of the disease is to be combated.

The above is also emphasized by Michielsen et al. (2010) who maintain that youth in developing countries are very susceptible to HIV infection and unintended pregnancy. The authors further state that this is caused by the experimental behaviour of young people, lack of knowledge, poverty, sociocultural factors and sex-inequality.

Youth are an important target in HIV and AIDS prevention programmes, as they are the future leaders. This view is also emphasized by HEAIDS (2010), which maintains that students are an important element of the population, as they are the future opinion makers who will have a great influence on policy. It is also important to focus on higher education students, given the fact that they are at risk as a result of the campus lifestyles, which may be conducive to actions that lead to HIV infections (HEAIDS, 2010). Universities, as institutions of higher learning, have a responsibility of safeguarding the well-being of students, and by implementing various interventions, including life skills training as a strategy to curb the spread of HIV and AIDS.

Sub-Saharan Africa carries an unreasonable burden of the world’s HIV and AIDS infections, and Ethiopia, Nigeria, South Africa, Zambia and Zimbabwe have the largest numbers of infections (UNAIDS, 2010). Southern Africa is still the most severely affected region in the continent. It is estimated that 11.3 million people are living with HIV and AIDS in Southern Africa. Putting this in a global context, 34% of people living with HIV and AIDS in 2009 were residing in Southern Africa, and 5.6 million of these people were living in South Africa (UNAIDS, 2010). This has grave implications for the country if efforts of preventing HIV and AIDS are not intensified.

The spread of HIV and AIDS has several implications for the population of South Africa. One of the areas in which the country is affected is at an economic level, as HIV and AIDS mostly affect economically active people between the ages of 15 and 49 years. The Association of African Universities (2004) highlights the importance of a focus on university students when it asserts that students are the primary stakeholders. The association further maintains that those programmes, which are offered to university students, should take into consideration the context of socio-economic factors that affect the students’ quality of life and their vulnerability to HIV and AIDS infection. This view is also confirmed by HEAIDS (2010) when it states that institutions of higher education should include HIV and AIDS education in their programmes. One of the benefits of life skills training is that it promotes assertiveness, and this may lower the risk of vulnerability to HIV infection.

The University of South Africa (2016) took a decision to integrate HIV/AIDS into the curriculum across all colleges and all levels of study, in order to produce HIV/AIDS competent graduates.

In response to the HIV and AIDS epidemic, the South African Departments of Education, Health and Welfare embarked on a national programme to implement life skills training, sexuality and HIV and AIDS education in secondary schools in 1995 (Department of Health and Department of Education, 1997/8).The goal of this intervention was to increase knowledge and skills needed for healthy relationships, effective communication and responsible decision-making that would protect learners from HIV infection. This training was also meant to promote a positive and responsible attitude towards people living with HIV and AIDS. The issue of life skills was more addressed in basic education than in higher education; hence, there is a need to assess the perception of students on life skills and HIV and AIDS prevention so as to come up with suggestions on how it can be infused in higher education settings. HEAIDS (2010) states that most graduates who started to work were found to lack the ability to deal with or manage HIV and AIDS in the workplace. This is an indication that higher education institutions did not prepare students to manage or deal with issues relating to HIV and AIDS.

The researchers, as staff members of a higher education institution, found it imperative to respond to the HEAIDS (2010) challenge referred to above and investigate if life skills could not be utilized as one way of assisting graduates to be able to deal with HIV and AIDS.

## Theoretical framework

The theory which was utilized to underpin this study was empowerment theory. Perkins and Zimmerman (1995) assert that empowerment is a concept that links individual strengths and competencies, natural helping systems, and proactive behaviours to social change.

According to Albertyn, Kapp, and Groenewald (2001), people possess power at various levels, namely, the micro level, interface level and macro level. The power that people possess at the micro level is related to the way a person feels about himself, including issues of dignity, self-confidence, self-esteem; leadership, coping skills, and assertiveness. This micro-level empowerment is related to life skills.

Empowerment is also linked to the choices that people have. Mohapi (2013) cites Alsop and Heinsohn (2005, p. 5) who view empowerment as enhancing an individual’s or group’s capability to make effective choices and convert these choices into actions and outcomes. This means that people have, through empowerment, power to decide which choices they make, and to take this further by exercising their choices to improve their conditions.

In the context of this study, empowerment means that students, through the acquisition of life skills, have the ability to make choices in situations where they are at risk of contracting HIV, and these choices will lead them to take action which will prevent them from contracting HIV.

## The importance of life skills

Life skills can be described as the ability for adaptive and positive behaviour that enables individuals to deal effectively with the demands and challenges of everyday life (World Health Organization, 1999). According to Visser (2005), life skills programmes focus on the development of various subsystems of the individual with the aim of enabling change in the person. For example, if a person changes the way he thinks, feels or takes decisions, this can lead to changes in the person’s behaviour. Because of this link in thinking, feeling, decision-making and behaviour, life skills training has the potential to have an impact on risk behaviour associated with HIV and AIDS.

In the context of the HIV and AIDS, the aim of life skills training is to develop young people’s knowledge, and the skills needed for healthy relationships, effective communication and responsible decision-making that will protect them and others from HIV infection and optimize their health (World Health Organization, 1993). In this regard, Visser (2005) maintains that it was found that life skills and HIV and AIDS education programmes for school children led to an increase in their knowledge of HIV and AIDS, they were more assertive, they displayed a positive attitude towards people infected with HIV, and there were some suggestions of delayed sexual activity.

Life skills are defined as skills necessary for successful living and learning (Loubser, 2012; Rooth, 2000). Life skills enhance the quality of life and prevent dysfunctional behaviours. Furthermore, Danish, Forneris, Hodge, and Heke (2004) define life skills as talents that can be learnt and used in daily life and that empower people to be successful in different environments. Life skills for students at a university include, amongst others, academic skills, career guidance, dealing with sexual and gender issues, skills to survive university and campus life and lifelong learning (HEAIDS, 2010). Therefore, given the issues raised previously regarding the context in which university students exist, serious thought should be given to the inclusion of life skills as a prevention strategy in the fight against the spread of HIV and AIDS. The concept of life skills, moreover, undertakes that individuals have access to the resources and power they need to change their lives.

HIV and AIDS prevention programmes that have balanced knowledge, attitudes and skills related to HIV transmission have proven more effective in actually altering behaviour than those that have concentrated on information alone. Life skills can improve HIV- and AIDS-related knowledge and self-awareness, and these positive effects can give rise to responsible citizens who are able to take responsibility for their own lives.

## Methodology

In the context of applied research, a quantitative study was undertaken, utilizing a survey as a non-experimental research design. This design was suitable for the study, as surveys have been found to be appropriate for exploratory, descriptive and explanatory and evaluative types of studies (De Vos, Strydom, Fouché, & Delport, 2011).

The research question which guided the study was: ‘What are the perceptions of Unisa students regarding life skills as a behaviour change strategy in the prevention of HIV and AIDS?’

The objectives of the study were:

1. to explore the perception of Unisa students regarding life skills as an HIV and AIDS prevention strategy,
2. to describe their needs regarding life skills as an HIV and AIDS prevention strategy and
3. to make recommendations regarding the content and presentation of a life skills programmes to promote the prevention of HIV and AIDS amongst students.

### Study site

Whereas South Africa, as a country, has nine provinces, the University of South Africa (Unisa) is divided into seven geographical regions in South Africa. These regions are Western Cape, Eastern Cape, Kwa-Zulu Natal, Gauteng, Limpopo, Mpumalanga and Midlands. The Midlands region in the Unisa context is a combination of the Northern Cape, Free State and North West Provinces of South Africa.

This study was conducted with University of South Africa students from three regions of the University, namely, Midlands, Gauteng and Limpopo regions. Student counsellors administered the questionnaires in these regions.

The sample from the Midlands region was collected in Kroonstad, Rustenburg and Mahikeng centres and which consisted of 36.8% of the participants. The Gauteng region sample was collected from Johannesburg, Ekurhuleni and Vaal and made up 36.8% of the participants. The sample from the Limpopo region was collected from Polokwane and Makhado centres and they made up 26.4% of the participants.

### Ethical considerations

The researchers obtained ethical clearance from the Department of Social Work at Unisa to allow them to conduct the research. The ethical clearance reference number is 1127896_05_13ꜞ. Permission to use students from regions was granted by the regional directors.

Furthermore, consent letters were given to all participants who took part in the study, explaining the purpose of the research and the method of data collection. The study participants were assured of anonymity, as they did not have to state their names or any identifying details on the questionnaire.

Confidentiality was ensured by storing the questionnaires in a place which was not accessible to third parties. Each questionnaire was accompanied by a blank envelope in which the participants had to put them and seal them.

Study participants were informed of the option of withdrawing from the study at any time when they felt threatened. Student counsellors from regional offices assisted in disseminating the questionnaires to students and finally submitting them back to the researchers for analysis.

### Study participants

Probability sampling was used in order to select students to participate in the study. Bryman (2012) asserts that when probability sampling is utilized, all elements in the population have an equal chance of being selected, and this also reduces sampling error.

The sample consisted of 334 Unisa students: 217 (65%) were female and 117 (35%) were male. The study level of the participants was represented as follows: 33% 1st level, 22% 2nd level, 23% 3rd level, 6% 4th level and 16% postgraduate students. The ages of participants varied from 18 to above 45 years.

The representation of students according to their ages were 18–25 (35.33%) of the sample, 26–35 (35.33%) 36–45 (21.26%) 45 years and above (8.08%).

The participants were sampled from the following colleges: College of Human Science 26.1%, College of Science, Engineering and Technology, 6.2%, College of Law 14.3%, College of Education 28.0%, College of Agriculture 3.4%, College of Economic and Management Sciences 22%.

### Questionnaire

A self-administered questionnaire was given to the participants. These questionnaires were given to the students by the student counsellors based in the respective regions, who acted as field workers. After the questionnaires were completed, they were returned to the researchers by the student counsellors.

The items in the questionnaire included the following: biographical data, awareness of HIV and AIDS and life skills training, need for knowledge on HIV and AIDS, need for intensification of HIV and AIDS awareness and facilitation requirements of life skills workshops.

In order to minimize bias and error, items which were not clear were eliminated from the questionnaire, and the instructions to the participants were standardized by means of a letter of instruction which was the same for all participants. The questionnaire was also piloted with a few students before being finalized (Strydom, 2011)

### Data analysis

Data were analysed statistically utilizing the Statistical Programme for Social Sciences to summarize, analyse and describe the research findings. Furthermore, the views of respondents were measured with ordinal scale type values, and this was done by calculating a mean score for the items that constitute each theme.

## Results

The results of the study focused mainly on the aspects that sought to explore the students’ perceptions of HIV and AIDS, awareness of HIV and AIDS and life skills training at Unisa, the need for HIV and AIDS, the need for the intensification of an awareness of HIV and AIDS at Unisa, frequency of life skills workshops at Unisa and facilitation requirements of life skills workshops at Unisa.

**Fig. 1.**
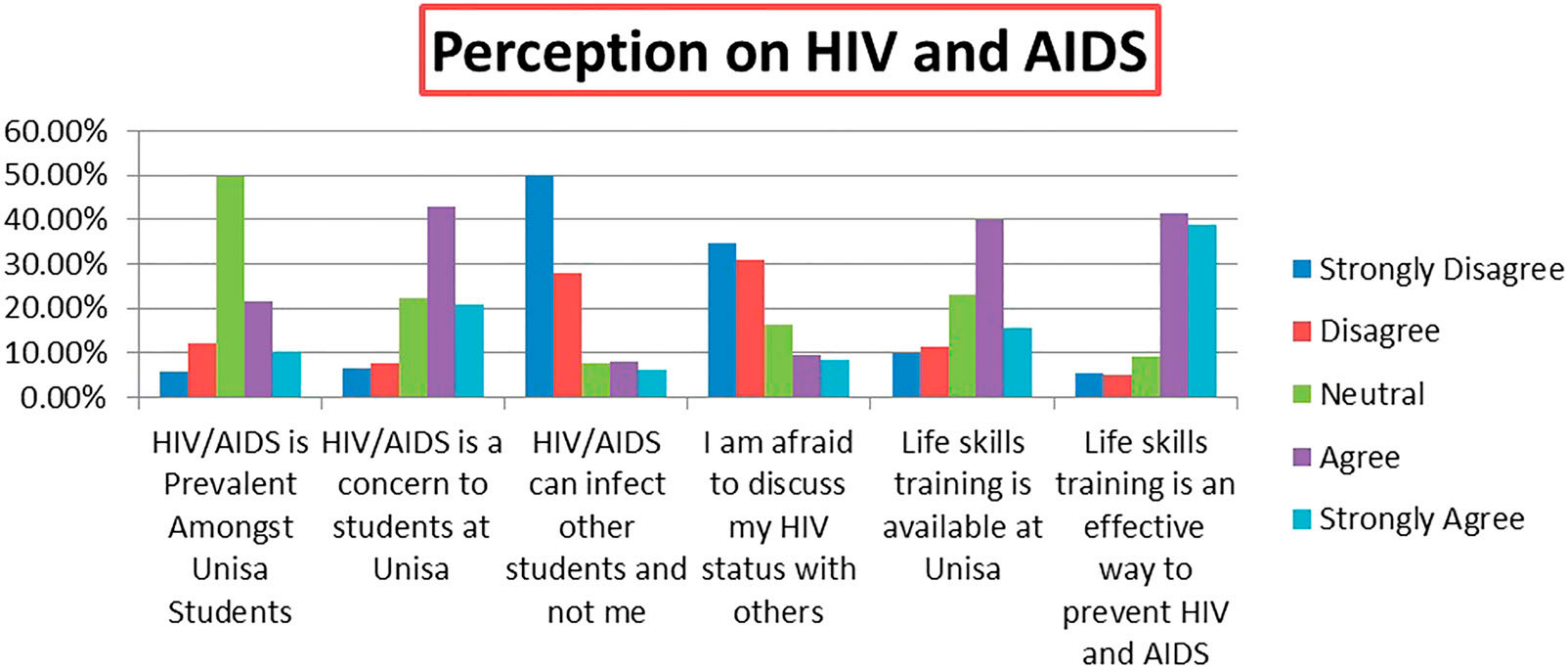
Perceptions of HIV and AIDS.

The results are summarized in Fig. 1.

In response to Section 1 of the questionnaire, the participants agreed and strongly agreed with item 1.6 (80.1%), which states that life skills training is an effective way of preventing HIV and AIDS. The respondents disagreed and strongly disagreed (78%) with item 1.3 which stated that HIV can infect other students and not them.

**Fig. 2.**
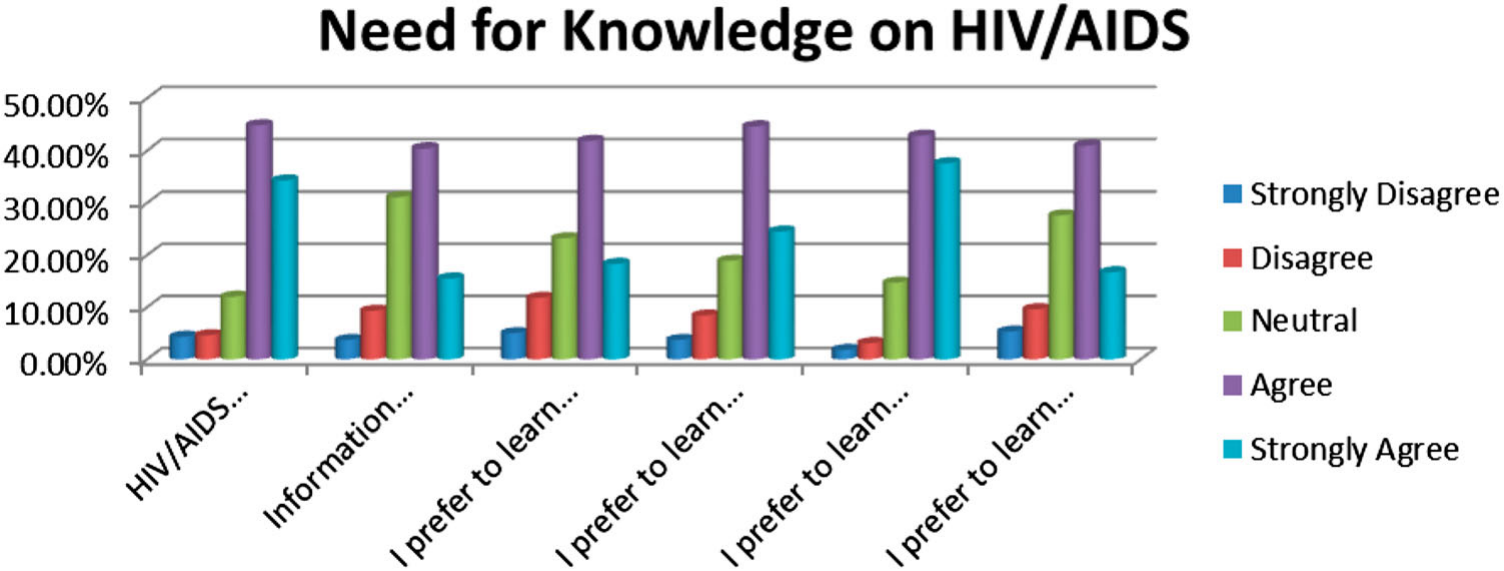
Need for knowledge on HIV and AIDS.

The notion that life skills training is effective in preventing HIV and AIDS is confirmed by Visser (2005) who states that there are indications that life skills programmes have a positive effect on the lives of young people. The importance of life skills programmes at the university level is also validated by HEAIDS (2010), which states that universities are not giving adequate attention to the issue of life skills and Aids in order to prepare them to be good citizens (Fig. 2).

This section focuses on the need for knowledge on HIV and AIDS.

The following items were included in this section:

(2.1) HIV/AIDS information should be integrated into the curriculum.
(2.2) Information sharing at Unisa on HIV and AIDS prevention is adequate.
(2.3) I prefer to learn about HIV and AIDS prevention by means of pamphlets.
(2.4) I prefer to learn about HIV and AIDS prevention by means of social media.
(2.5) I prefer to learn about HIV and AIDS prevention through life skills workshops.
(2.6) I prefer to learn about HIV and AIDS prevention by getting information in newspapers.
(2.7) I prefer to learn about HIV and AIDS prevention from my family.

The study participants agreed and strongly agreed with items 2.1 (79.1%), and 2.5 (80.4%). The respondents disagreed and strongly disagreed by (17.1%) and neutral (22.3%) with item 2.7 that they prefer to learn about HIV and AIDS from their family. This may be attributed to the sensitivity and stigma around issues of HIV and AIDS, and this may inhibit open communication in some families.

The choice of life skills as a preventative strategy is supported by Visser (2005) who states that there is growing proof that life skills programmes have a positive influence on the lives of children and adolescents. This view is also emphasized by HEAIDS (2010) which expresses the view that life skills training has the potential to contribute to HIV and AIDS prevention, but this is not properly utilized in most universities.

**Fig. 3.**
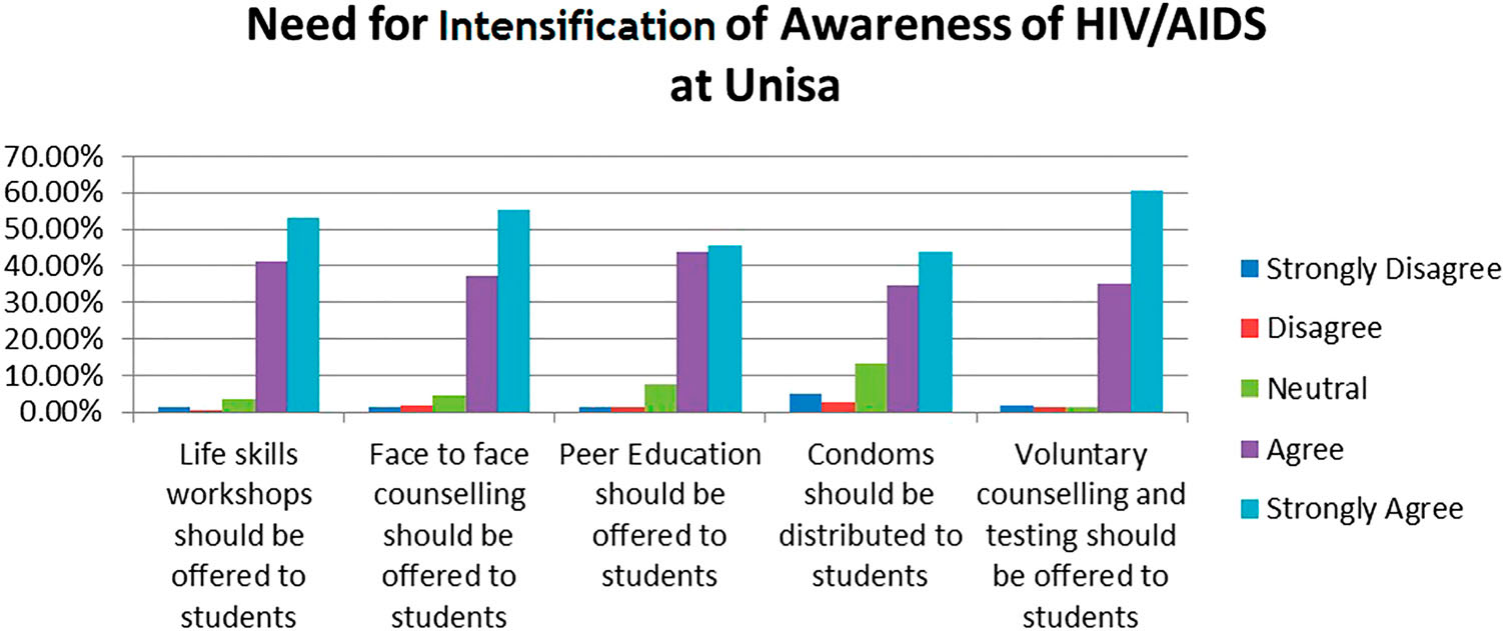
Need for intensification of awareness of HIV and AIDS at Unisa.

Figure 3 reflects the findings on the statements posed regarding students’ needs for an intensification of an awareness of HIV and AIDS at the University of South Africa. In the above figure, the study participants agreed and strongly agreed (with 95.7%) with item 3.5, which states that voluntary counselling and testing should be offered to students. The second-highest level of agreement, which is 94.4%, is about the offering of life skills to students. The third-highest level of agreement, which is 78.9%, was for item 3.4, which is about condom distribution as a means of preventing HIV and AIDS. These agreement levels confirm that the practice of voluntary counselling and testing should continue, and that life skills workshops should be added as a means of intensifying the prevention of HIV and AIDS amongst students. HEAIDS (2010) confirms this view by stating that there is a role to be played by universities in dealing with HIV and AIDS. One of these methods could be the offering of life skills workshops, as students indicate that there is a need for such workshops as a behaviour change strategy for the prevention of HIV and AIDS.

**Fig. 4.**
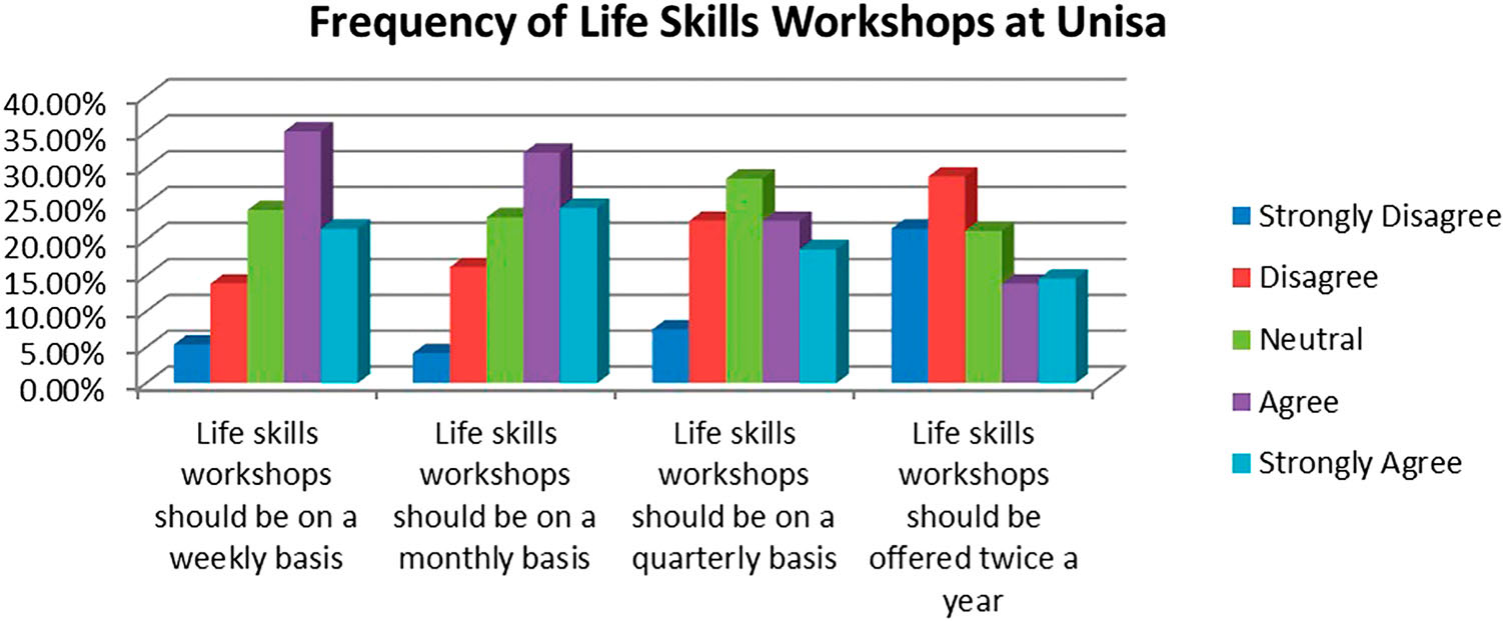
Frequency of life skills workshops at Unisa.

Figure 4 indicates the students’ views on the frequency of offering life skills workshops at Unisa. Here, the highest level of agreement is where the participants agree and strongly agree (56.6%) with the statement that life skills workshops should be held on a weekly basis.

The method of teaching life skills through workshops is also known as an add-on or adjunct approach, as these life skills are not integrated into mainstream curricula. This method of offering life skills is emphasized by De Jong, Lazarus, Ganie, and Prinsloo (1995) who maintain that particular developmental needs and contextual demands will not be adequately met by a life skills curriculum which is infused entirely into the general curriculum, like AIDS education, which would require a separate space in the curriculum.

According to HEAIDS (2010), life skills for university students include academic skills, career guidance, dealing with sexual and gender issues and skills to survive campus and university life. All these skills can be offered in an interactive workshop format.

**Fig. 5.**
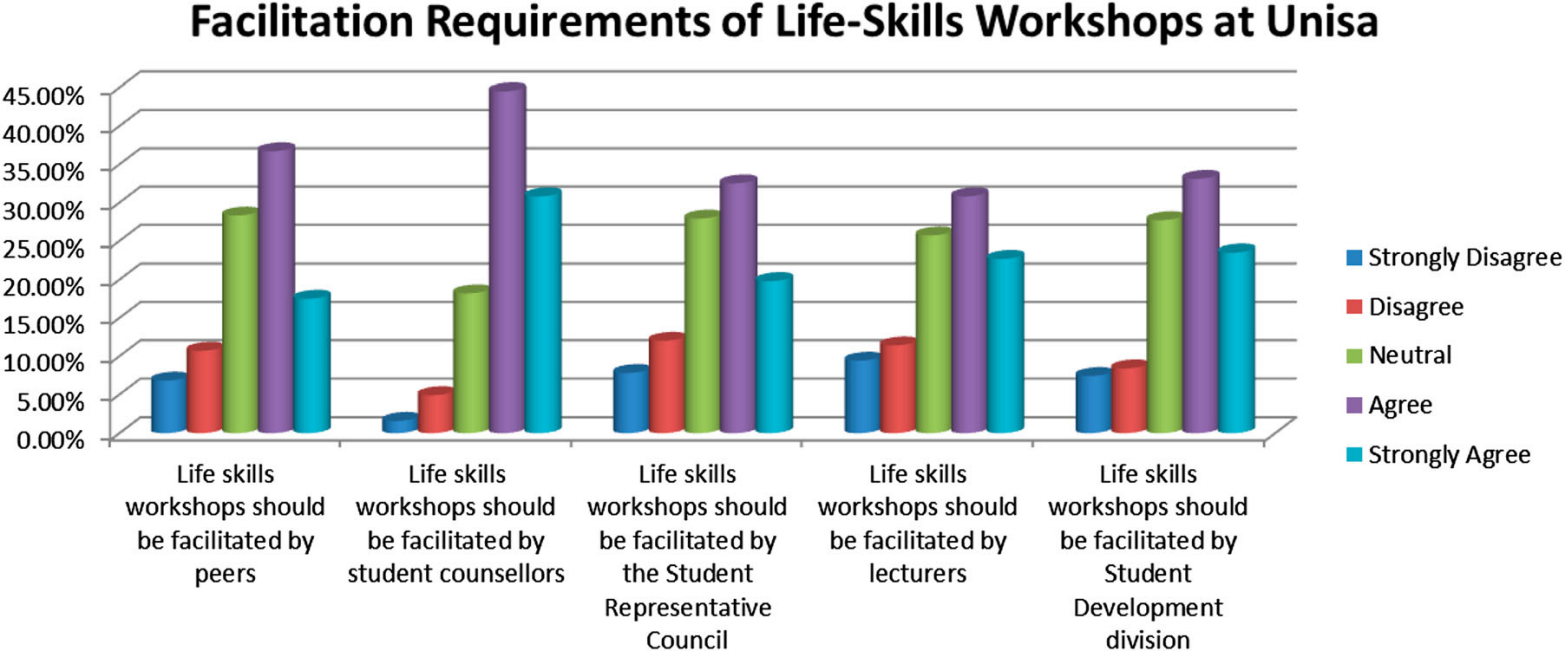
Facilitation requirements of life skills workshops at Unisa.

Figure 5 focuses on the question regarding facilitation requirements of life skills workshops at Unisa. In the above table, the respondents agreed and strongly agreed (75.3%) with the statement that life skills workshops should be facilitated by student counsellors. The respondents disagreed and strongly disagreed (20.8%) with the statement that life skills workshops should be facilitated by lecturers.

Rooth (1995) asserts that life skills facilitators have to have knowledge of group processes and dynamics. Student counsellors, most of whom have studied psychology, will have the necessary knowledge on group dynamics.

The above view endorses the findings of HEAIDS (2010), which states that life skills training should be done by professional staff who are committed to doing this training. Mutinta, Govender, Gow, and George (2012) also state that HIV prevention initiative in universities should include educating students using comprehensive models. This fits in very well with the notion of including life skills training as one of the ways of preventing the spread of HIV and AIDS.

## Discussion

The results of the study indicate that life skills training is perceived to be an effective way of preventing HIV and AIDS. Most students are of the opinion that through life skills training they will reduce the risks of infections and re-infections, as they will be equipped with knowledge, which will assist them in taking care of themselves. This will further minimize the fear of stigma and deep-rooted discrimination surrounding HIV issues, which makes young people less likely to adopt preventative strategies such as using condoms, testing for HIV and other STIs, and adhering to treatment or disclosing their HIV status to sexual partners as highlighted in UNAIDS (2002).

The study revealed that students have knowledge about HIV and AIDS preventative measures, but this does not prevent them from needing more assistance regarding further interventions on taking care of themselves through activities like life skills workshops. The workshops may reduce the stigma surrounding HIV and AIDS, as students will have an opportunity to share their views openly with each other. In this regard, Yankah and Aggleton (2008) maintain that with regard to HIV and AIDS, life skills are seen as being able to make possible the negotiation of vulnerability and risk. They permit open communication about sex and other issues. This is important for young people in order to promote the prevention of HIV and AIDS.

As far as HIV and AIDS knowledge is concerned, the study participants indicated that they preferred to learn about HIV and AIDS prevention through life skills workshops rather hearing information from their families. The reason for this may be that they will feel comfortable sharing their ideas and concerns with their peers rather than with close family members who may reject them or be sympathetic instead of accepting and assisting them to deal with the issue at hand.

The above is confirmed by a study conducted by Dunbar et al. (2010), who found that life skill sessions were said to be very popular and providing support amongst young people. The authors further state that ‘participants gained a sense of personal value and hope, as well as skills to communicate effectively and assertively, including learning to say and mean no’ (Dunbar et al., 2010). This is a very important skill in HIV and AIDS prevention, as life skills have the potential to teach young people assertiveness.

Regarding the need of intensification of HIV and AIDS awareness campaigns, most of the participants stated that more voluntary counselling and testing should continue to try and strengthen the awareness of HIV and AIDS among students at universities.

The questions that dealt with the frequency of life skills workshops revealed that many students would prefer to have workshops facilitated once per week as opposed to being offered daily. Facilitation of workshops weekly would remind students of the responsibilities they have in their lives as stated by Msila (2007). Furthermore, Visser (1996) suggests that life skills training contributes to increased levels of knowledge regarding HIV and AIDS, more assertiveness and a more positive attitude towards people living with HIV and AIDS. This is a clear indication that the frequency of life skills workshops is very important, as it can help promote a positive attitude amongst university students.

In response to the questions on facilitation of life skills workshops, the study participants prefer that facilitation of workshops should be done by student counsellors. This is in line with HEAIDS (2010) which states that life skills are mainly facilitated by peer educators at many universities, and life skills training can only be effective if conducted by professional staff. Student counsellors are professional social workers and psychologists.

From the discussion above, it can be concluded that most students have a need for an intensification of HIV and AIDS awareness campaigns, which can be done through weekly life skills workshops.

## Limitations of the study

The study was conducted amongst Unisa students. Accordingly, students at other institutions of higher learning may have different experiences and perceptions, and this may limit the generalization of the findings.

Although the sample of 334 may be deemed to be large in a quantitative study, it is a limited sample if you take into consideration the size of the institution from which the sample was drawn.

## Recommendations

Students at Unisa strongly feel that life skills training should be offered as a behaviour-changing strategy, and that these workshops should be facilitated by student counsellors. The frequency of workshops could be on a weekly basis, depending on the mode of offering. Unisa offers Open and Distance Learning and an opportunity exists for innovation with regard to the offering of these workshops. Workshops may be facilitated at the various learning centres, or they may be offered online, to reach students who are not in a position to travel to the learning centre to attend the workshops. An array of media could also be used to conduct workshops or to disseminate information, for example, YouTube, video recordings or video conferencing.

## Conclusion

The study has revealed that students have a need for life skills workshops as a means of changing behaviour in order to strengthen the prevention of HIV and AIDS. Furthermore, an opportunity exists for Unisa to be innovative by offering the workshops in different modes, that is, face-to-face, online or by means of other media. Student counsellors should be involved in offering these workshops in addition to other efforts already initiated by the university to prevent HIV and AIDS, such as peer counselling, condom distribution and HIV/AIDS curriculum integration.
